# C‐Reactive Protein Serum Values in Idiopathic and Autoimmune Subglottic Stenosis

**DOI:** 10.1002/lary.70167

**Published:** 2025-09-24

**Authors:** Andrew J. Neevel, Fatemeh Ramazani, Julia Ford, Ora Gewurz‐Singer, Norman D. Hogikyan, Robert J. Morrison, Robbi A. Kupfer

**Affiliations:** ^1^ Department of Otolaryngology‐Head and Neck Surgery University of Michigan Ann Arbor USA; ^2^ Department of Internal Medicine, Division of Rheumatology University of Michigan Ann Arbor USA

**Keywords:** c‐reactive protein, granulomatosis with polyangiitis, prognostic factors, recurrence, subglottic stenosis

## Abstract

**Objective (s):**

Idiopathic and granulomatosis with polyangiitis (GPA)‐related subglottic stenosis (SGS) are considered distinct immune‐mediated inflammatory disorders. Limited data exist on serum inflammatory markers, specifically C‐Reactive Protein (CRP), for SGS diagnosis, differentiation, and prognostication. The study objective was the characterization of CRP levels in idiopathic (iSGS) and GPA‐SGS to assess CRP's ability to distinguish SGS subtypes and predict SGS progression and recurrence.

**Methods:**

Retrospective review of patients with idiopathic or GPA‐SGS from 2007 to 2024 at a single institution. Surgery‐free interval (SFI) was calculated as the time between surgical interventions. Statistical analysis included independent t‐tests, chi‐squared, and univariate linear regression.

**Results:**

59 iSGS and 47 GPA‐SGS patients were included. CRP was elevated in 36% of iSGS and 49% of GPA‐SGS patients (0.0.6 mg/dL). Mean maximum CRP was 2.7 mg/dL lower in iSGS compared to anti‐neutrophil cytoplasmic antibody (ANCA)‐positive GPA‐SGS (*p* = 0.035). The maximum CRP in iSGS was 3.6 mg/dL, while 10 (21%) GPA patients had CRPs greater than 3.6 mg/dL (max = 31 mg/dL). ANCA‐negative GPA‐SGS mean CRP was not significantly different than iSGS or ANCA‐positive GPA‐SGS. CRP and SFI did not correlate on univariate linear analysis.

**Conclusion:**

Mild elevation of CRP is common in SGS patients. High CRP levels are more frequent in GPA‐SGS, potentially aiding clinical differentiation of etiologies. However, CRP at presentation does not appear to correlate with disease recurrence in iSGS or GPA‐related SGS, limiting its value as a biomarker and prognostic tool.

**Level of Evidence:**

3

## Graphical abstract

This study characterized C‐reactive protein (CRP) levels in idiopathic and granulomatosis with polyangiitis‐related (GPA) subglottic stenosis (SGS) to assess CRP ability to distinguish SGS subtypes and predict recurrence. High CRP levels are more frequent in GPA‐SGS, potentially aiding clinical differentiation of etiologies. CRP at presentation does not appear to correlate with disease recurrence in iSGS or GPA‐SGS, limiting its value as a biomarker and prognostic tool.

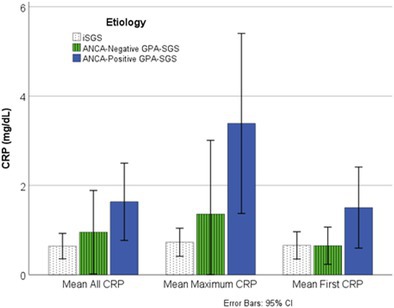

## 1 Introduction

Idiopathic subglottic stenosis (iSGS) is airway narrowing believed to be secondary to an epithelial‐to‐mesenchymal transition, occurring without an obvious inciting event, in the subglottis [1]. Contemporary literature attributes this disease's transitional process to a dysregulated immune response in the subglottic mucosa, causing collagen and extracellular matrix deposition [1]. Various studies have demonstrated the role of several cytokine‐signaling pathways in the propagation of the inflammatory response leading to iSGS [2, 3, 4, 5]. While these studies demonstrate the role of immune dysregulation in vitro and through tissue biopsies, there is little current data assessing the use of serum inflammatory markers for the diagnosis and prognostication of iSGS. One fibrotic serum biomarker, S100A8/A9, was recently found to correlate with surgery‐free interval (SFI), the time between endoscopic procedures and a clinically relevant measure of disease recurrence [6]. However, no serum test is currently available for clinical use. Serum testing may pose the inherent disadvantage of distance from the affected tissue, but it is an easily accessible, readily available, potential diagnosis and prognostic tool.

Granulomatosis with polyangiitis, a systemic inflammatory disease, is another cause of SGS that often presents a diagnostic challenge for otolaryngologists and rheumatologists. GPA may be isolated to the subglottis [7]. Idiopathic SGS was initially thought to be an isolated phenotype of GPA‐SGS, but growing evidence suggests that iSGS is a separate pathophysiologic process [8, 9, 10]. Differentiation between GPA‐SGS and iSGS strongly influences clinical management, especially in consideration of systemic therapy [11]. Anti‐neutrophil cytoplasmic antibodies (ANCA) are positive in approximately 85% of endobronchial GPA patients, which leaves a subset of patients with airway manifestations and no definitive GPA diagnosis [7].

C‐reactive protein (CRP) is an acute phase reactant whose transcription is thought to be induced by interleukin‐6 (IL‐6) during inflammatory and infectious processes [12]. Compared to erythrocyte sedimentation rate (ESR), CRP is a more direct test for inflammation and is thought to rise and fall rapidly with the introduction and removal of an inflammatory stimulus [12]. This positions CRP as a useful diagnostic and potentially prognostic tool. Additionally, the presence of IL‐6 as a biomarker in mucosal surfaces of patients with iSGS may lead to a possible elevation of CRP levels with active disease in this patient population [3]. A recent study by Sun et al. identified CRP as a possible prognostic factor for interventional therapeutic outcomes in central airway stenosis [13]. However, there are no studies in the literature assessing the role of CRP for the diagnosis and long‐term prognostication of SGS.

CRP had been shown to be an independent predictor for overall survival in ANCA‐associated vasculitis (AAV), which includes GPA [14]. CRP is often elevated in GPA, especially during inflammatory flares or widespread systemic disease, but non‐specific elevation complicates isolated diagnostic and prognostic utility. It is our center's practice to order a panel of autoimmune blood tests during initial subglottic stenosis workup. These include CRP, ESR, anti‐nuclear antibody (ANA), and anti‐neutrophil cytoplasmic antibody (ANCA). If CRP was shown to differ reliably in varying aggressiveness of iSGS and GPA‐SGS, it could provide a cost‐effective approach to diagnosis, prognostication, and clinical decision‐making.

The objective of this study was to compare CRP levels in iSGS and GPA‐SGS and evaluate its utility in distinguishing these subtypes and predicting disease progression and/or recurrence.

## 2 Materials and Methods

### 2.1 Patient Population

This study was reviewed and determined to be exempt by the Institutional Review Board at the University of Michigan (HUM00245703). Informed consent was not required. DataDirect, an institutionally hosted medical record search tool, was used to retrieve all patients treated at the University of Michigan from January 2007 to October 2024 who met the study criteria: those diagnosed with idiopathic or GPA‐related subglottic stenosis with available inflammatory lab results. Patients with subglottic stenosis were screened using International Classification of Diseases, 9th edition (ICD‐9) and International Classification of Diseases, 10th edition (ICD‐10) codes for laryngeal stenosis (519.1, 519.19, 478.74, J38.6, J38.7, J39.8, and J95.5). These patients were then filtered for ages greater than 18 years old and availability of CRP, ESR, ANA, or procalcitonin serum laboratory data. An institution‐hosted text‐based search tool, Electronic Medical Record Search Engine (EMERSE), was then used to screen patients individually for idiopathic subglottic stenosis and GPA‐related SGS diagnoses. Patients with iSGS were excluded if they had concurrent rheumatologic diagnoses that could increase serum inflammatory factor levels. Patients were considered to have GPA‐SGS if they had a definitive diagnosis of both GPA by rheumatology and SGS by laryngology. ANCA‐negative GPA‐SGS patients were biopsy‐positive or clinically diagnosed by rheumatology with the presence of other systemic manifestations.

### 2.2 Exposures and Outcomes

Demographic data including age, sex, race/ethnicity, relevant comorbidities, and SGS subtype were collected. Endoscopic procedure dates for balloon dilation and cold/laser excision were collected. Surgery‐free interval (SFI) was calculated as the time between endoscopic procedures, which required at least 2 procedure dates. History of in‐office serial intralesional steroid injections and timing of immunosuppressive agents (rituximab, cyclophosphamide, or methotrexate) were also recorded. Relevant laboratory data (test, date, and value) for each patient were pulled using DataDirect. A normal CRP value ranges from 0.0 to 0.6 mg/dL for our institutional assay. CRP values were included if drawn as an outpatient or upon presentation to the emergency department. “First CRP” was defined as CRP at first presentation to our institution. “CRP at diagnosis” was defined as CRP at the time of definitive clinical diagnosis. Presentations with GPA flares were included, but isolated upper respiratory infections or pneumonia cases were excluded. CRP values from daily laboratory data from inpatient hospitalizations were excluded. CRP values were considered “on immunosuppression” if the patient was actively taking methotrexate or cyclophosphamide or within 1 year of a rituximab dose [15].

### 2.3 Data Analysis

The frequencies of elevated CRP were compared between iSGS, ANCA‐negative GPA‐SGS, and ANCA‐positive GPA‐SGS using Chi‐squared analysis. Average CRP was calculated by patient to evenly weight CRP value representation, and then means were compared between iSGS, ANCA‐negative GPA‐SGS, and ANCA‐positive GPA‐SGS patients using one‐way ANOVA. Dunnett's T3 post hoc analysis was used due to sample size < 50 patients, with no assumed equal variance.

Mean CRP on first evaluation at our institution (“First CRP”), mean CRP at the time of definitive diagnosis, and mean maximum CRP were also compared between iSGS, ANCA‐negative GPA‐SGS, and ANCA‐positive groups using one‐way ANOVA. CRP values across immunosuppression and potential confounding comorbidities (obesity, smoking, hypertension, coronary artery disease, and diabetes) were compared using independent t‐tests. Univariate linear regression using normalized log_10_ (CRP) value was used to compare the change in CRP and SFI in both iSGS and GPA‐SGS separately. Data analysis was performed in SPSS Statistics 29.0.0.0 (IBM).

## 3 Results

The initial ICD code search for non‐specific laryngeal stenosis with filters for age > 18, treatment by our institution's laryngologists, and available inflammatory lab values yielded 295 total patients. Manual review with EMERSE identified 106 patients with either iSGS (*n* = 59, 56%), ANCA‐negative GPA‐SGS (*n* = 10, 9%), or ANCA‐positive GPA‐SGS (*n* = 37, 35%). Twenty‐nine iSGS patients (49%) and 19 GPA‐SGS patients (40%) had multiple OR dates and therefore had calculable SFIs. Idiopathic SGS patients were 100% white and 100% female. GPA‐SGS patients were also predominantly female and white (Table 1). Twenty‐four iSGS patients (41%) and 7 GPA‐SGS patients (15%) underwent serial intralesional steroid injections (SILSI).

**TABLE 1:** Demographics and comorbidities.

	iSGS, (*n* = 59)	ANCA‐negative, GPA‐SGS (*n* = 10)	ANCA‐positive GPA‐SGS (*n* = 37)	All, (*n* = 106)
Mean age at presentation in years (range)	53 (22, 76)	49 (31, 82)	44 (17, 75)	50 (17, 82)
Sex, *N* (%)				
Male	0 (0%)	0 (0%)	11 (30%)	11 (10%)
Female	59 (100%)	10 (100%)	26 (70%)	95 (90%)
Race, *N* (%)				
White/Caucasian	59 (100%)	10 (100%)	34 (92%)	103 (97%)
Hispanic/Latino	0 (0%)	0 (0%)	2 (5%)	2 (2%)
Black/African‐American	0 (0%)	0 (0%)	1 (3%)	1 (1%)
Obesity at presentation, *N* (%)				
Not obese (< 30 kg/m^2^)	29 (49%)	5 (50%)	21 (57%)	55 (52%)
Obese (≥ 30 kg/m^2^)	30 (51%)	5 (50%)	16 (43%)	51 (48%)
Smoking, *N* (%)				
Never	53 (90%)	9 (90%)	27 (73%)	89 (84%)
Former	6 (10%)	1 (10%)	9 (24%)	16 (15%)
Active	0 (0%)	0 (0%)	1 (3%)	1 (1%)
Comorbidities, *N* (%)				
Diabetes	9 (15%)	3 (30%)	6 (16%)	18 (17%)
Hypertension	21 (36%)	2 (20%)	15 (41%)	39 (37%)
Coronary Artery Disease	2 (3%)	0 (0%)	3 (8%)	5 (5%)
Congestive Heart Failure	1 (2%)	0 (0%)	1 (3%)	2 (2%)

Abbreviations: ANCA, anti‐neutrophil cytoplasmic antibody; GPA‐SGS, granulomatosis with polyangiitis related subglottic stenosis, iSGS, idiopathic subglottic stenosis.

**FIGURE 1:**
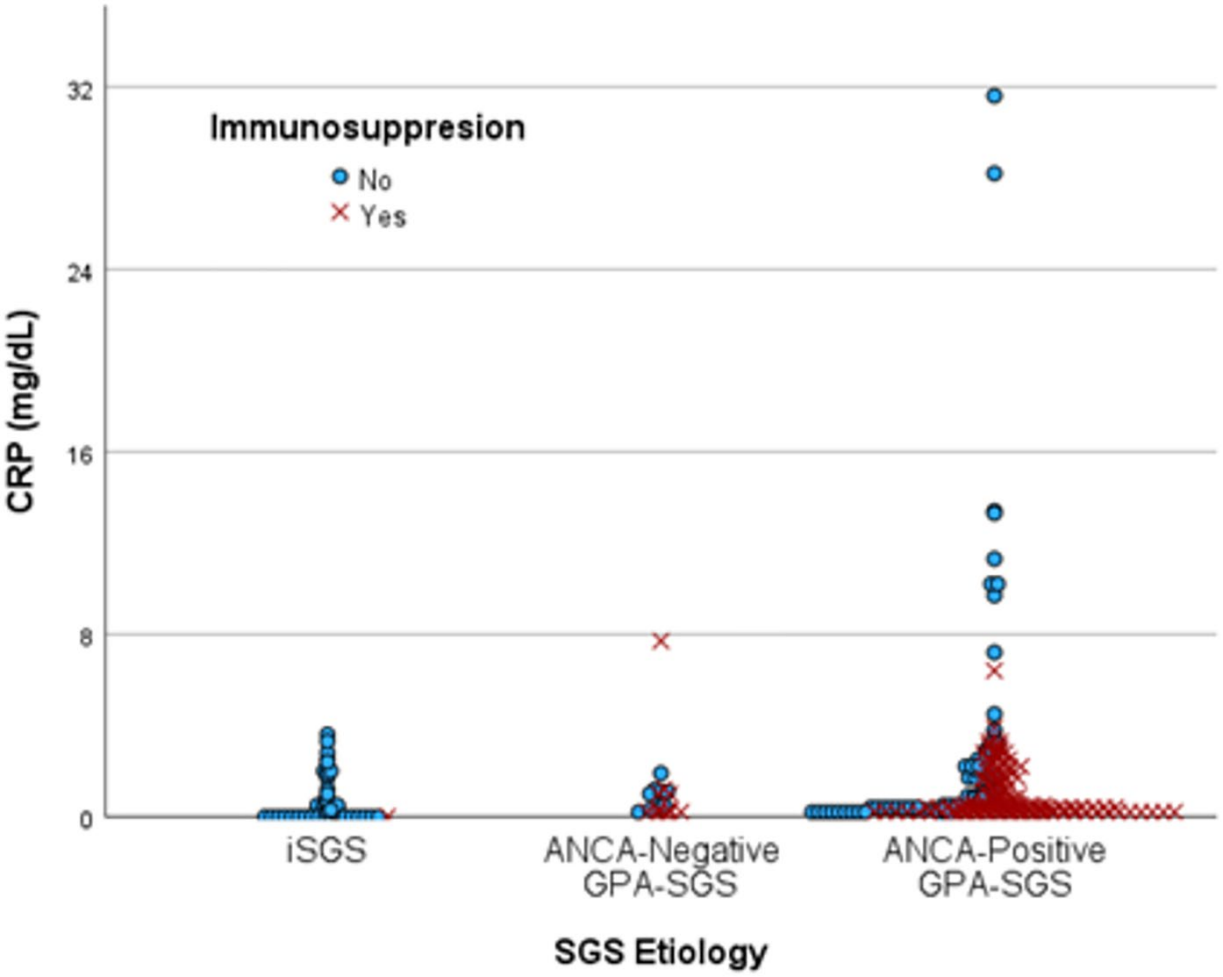
All CRP values compared by subglottic stenosis etiology and immunosuppression status. CRP, C‐reactive protein; GPA, granulomatosis with polyangiitis. CRP Values = mg/dL. [Color figure can be viewed in the online issue, which is available at www.laryngoscope.com]

**FIGURE 2:**
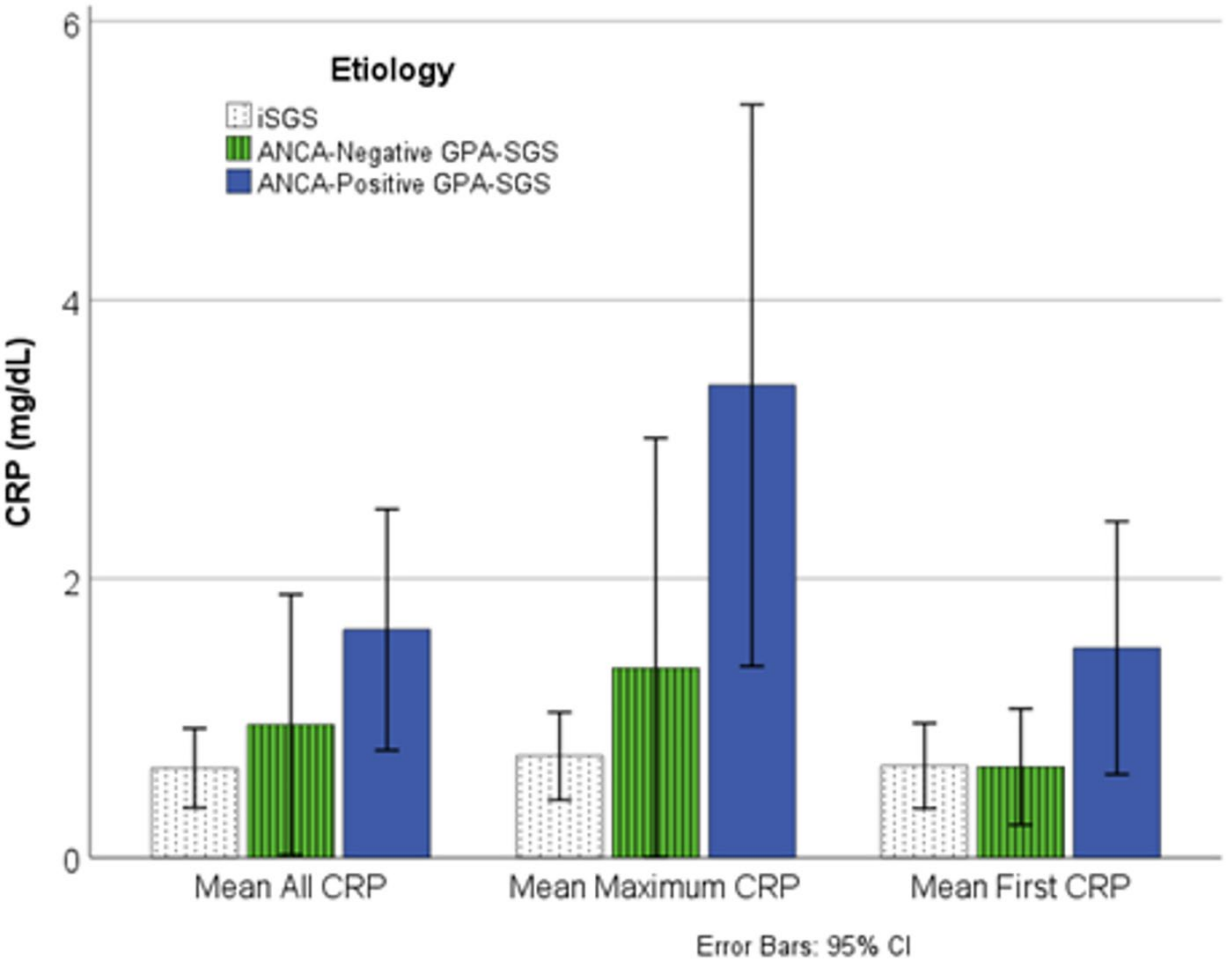
Mean CRP by patient, maximum CRP, and CRP at first presentation to our institution in iSGS, ANCA‐negative GPA‐SGS, and ANCA‐positive GPA‐SGS. * = *p* < 0.05, ** = *p* < 0.01. CI, confidence interval; CRP, C‐reactive protein; GPA, granulomatosis with polyangiitis; SGS, subglottic stenosis; CRP Values = mg/dL. [Color figure can be viewed in the online issue, which is available at www.laryngoscope.com]

**FIGURE 3:**
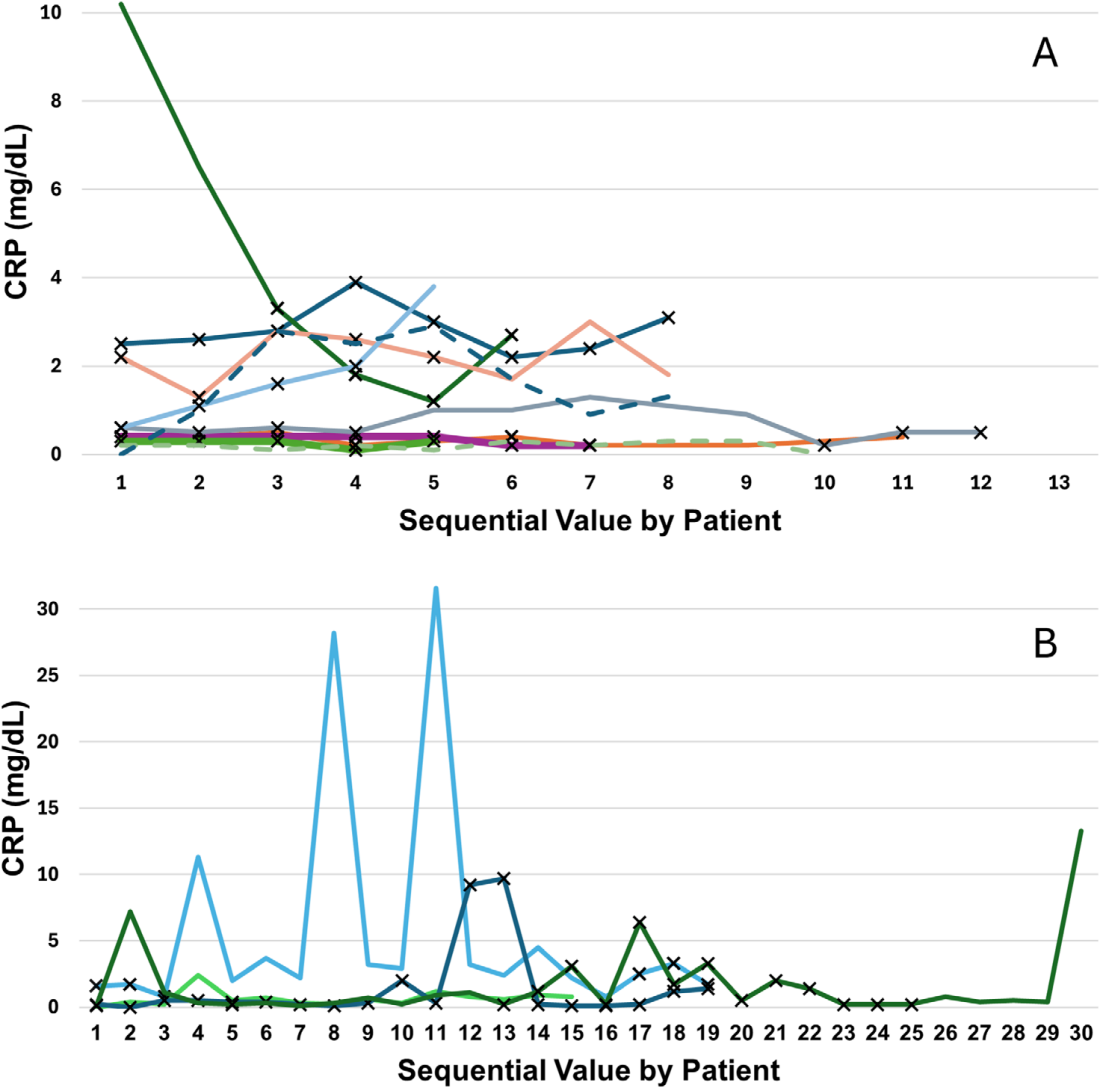
Sequential CRP values of GPA‐SGS patients with more than 5 values available. Patients divided into panels A and B based on scale. X indicates active immunosuppression. Dotted lines indicate ANCA‐negative status. CRP, C‐reactive protein. [Color figure can be viewed in the online issue, which is available at www.laryngoscope.com]

A total of 39 CRP values were available in 59 iSGS patients, and 223 CRP values were available in 47 GPA‐SGS patients (Figure 1). Ninety‐two percent of iSGS patients had a single CRP drawn at presentation to a laryngologist at our institution, which was often at the time of new diagnosis. Including all timepoints, CRP was elevated (> 0.6 mg/dL) in 36% of iSGS, 40% of ANCA‐negative GPA‐SGS, and 51% of GPA‐SGS patients. Mean CRP by patient was 1 mg/dL lower (*p* = 0.058) in iSGS (0.6 mg/dL ±0.28 mg/dL) compared to ANCA‐positive GPA‐SGS (1.6 mg/dL ±0.86 mg/dL) (Figure 2). Mean maximum CRP was 2.7 mg/dL lower in iSGS compared to ANCA‐positive GPA‐SGS (*p* = 0.035). The maximum CRP in iSGS was 3.6 mg/dL, while eight ANCA‐positive GPA‐SGS patients had CRPs greater than 3.6 mg/dL (max = 31.6 mg/dL). Two ANCA‐negative patients had CRPs greater than 3.6 mg/dL (max = 7.7 mg/dL) (Figure 3A,B). Mean CRP at first presentation to our institution was not significantly different between iSGS (0.7 mg/dL) and ANCA‐positive GPA‐SGS (1.1 mg/dL) patients. CRP at new diagnosis was only available in 17% of GPA‐SGS patients, but the average was significantly different from iSGS (*p* = 0.01). All CRP comparisons, including the 10 ANCA‐negative GPA patients, were not significantly different from the other two groups.

CRP and SFI did not correlate on univariate linear analysis as an entire cohort and when separated into iSGS and GPA‐SGS groups. There was also no correlation between body‐mass index (BMI) and CRP on univariate linear analysis. Potential confounding comorbidities, including obesity (> 30 kg/m^2^ BMI), smoking, hypertension, coronary artery disease, and diabetes, did not have a significant effect on mean CRP by patient, maximum CRP, or CRP at first presentation when compared with independent *t*‐tests. GPA‐SGS CRP values drawn during immunosuppression (active methotrexate, active cyclophosphamide, or recent rituximab treatment) had a 1.7 ± 0.9 mg/dL lower mean CRP (*p* < 0.01).

## 4 Discussion

We performed an in‐depth analysis of serum CRP levels across a cohort of iSGS and GPA‐SGS patients. This work demonstrated that a third of patients with iSGS have mild CRP elevation (> 0.6 mg/dL). In ANCA‐positive GPA‐SGS, CRP was more commonly elevated (50%) and included eight patients with CRP levels > 3.6 mg/dL, the iSGS maximum value. CRP values were lower (mean 1.7 mg/dL) with exposure to immunosuppression. Common comorbidities that may elevate baseline CRP did not significantly impact CRP levels. CRP did not correlate with surgery‐free interval, a proxy for disease severity [16].

This work demonstrates the value of serum CRP as a contributor to the diagnosis of SGS and differentiation between iSGS and GPA‐SGS. Low or mildly elevated CRP is common in both iSGS and GPA‐SGS and therefore cannot be used to differentiate between SGS subtypes. Patients with highly elevated CRP (> 3.6 mg/dL) and no other apparent inflammatory process apparent are far more likely to have GPA, as no iSGS patients had a CRP > 3.6 mg/dL. However, the sensitivity is limited as half of GPA‐SGS patients had normal CRP levels on presentation. Our sample size of 10 ANCA‐negative patients limited comparison, but its mean CRP of 0.95 mg/dL and 20% of patients with > 3.6 mg/dL suggest a process with greater inflammatory activity than iSGS but potentially less systemic burden than ANCA‐positive GPA.

A single study from France described CRP levels in a smaller cohort of acquired subglottic and tracheal stenosis patients and also found that CRP elevation was more frequent in GPA and relapsing polychondritis (RP) than iSGS [17]. The common normal or mildly elevated CRPs in GPA patients in our study are similar to data‐driven clustering of GPA phenotypes. Clusters of young patients with isolated manifestations of ear, nose, and throat were found to have a median CRP of 0.6 mg/dL, versus a mean CRP of 5.7 mg/dL across all GPA patients [18]. The high percentage of normal or mildly elevated CRP values in our GPA‐SGS participants further supports the importance of a complete autoimmune workup including a laboratory panel and consideration of a rheumatology referral in newly presenting isolated airway stenoses.

CRP values are highly variable and responsive to systemic inflammation and infection. We collected common conditions that may increase baseline serum CRP, including obesity, hypertension, congestive heart failure, coronary artery disease, and smoking [12, 19]. All patients who were former smokers had a remote history > 9 years, so no current effect on CRP was expected [20]. These factors did not have a statistically significant impact on serum CRP in our cohort, though they may introduce underlying variability. CRP values associated with diagnosed acute infectious processes were excluded, but mild elevations in CRP values could have been related to upper respiratory infections [21]. Any interpretation of CRP should involve comprehensive consideration of a patient's comorbidities, lifestyle, and acute changes to health. Immunosuppression was an expectedly relevant effector of CRP in GPA‐SGS patients. However, there were still large (> 1 mg/dL) fluctuations in CRP values both on and off immunosuppression, which could correspond to disease activity and inform multidisciplinary clinical decision‐making.

Our findings also show that single CRP on presentation does not have prognostic value for overall disease aggressiveness, as CRP did not correlate with mean SFI in both GPA‐SGS and iSGS. This is unsurprising given the variability of single CRP values, which can change based on diet, infection, and many other individual factors [22, 23]. Elevated CRP was associated with long‐term therapeutic failure in scarring central airway stenosis, but it is unclear at what timepoints these values were measured [13]. There remains a gap in prognostic testing for SGS, which could add to endoscopic exam findings, patient symptoms, and peak expiratory flow [16, 24]. Quantification of inflammatory burden and risk of rapid recurrence could inform treatment decision‐making, including systemic therapy, timing and course of serial intralesional steroid injections, and endoscopic surgery timing. Further, laboratory serum tests could expand access beyond tertiary care centers, reducing costs for medical systems and patients. Significant progress has been made on this front, including the characterization of serum S100A8/A9 and mucosal PMEPA1 which correlated with SFI [6, 10]. Nonetheless, there is not an easily accessible, cost‐effective prognostic test for SGS progression and decision‐making.

As discussed above, our sample size of ANCA‐negative patients limited our ability to assess if CRP can reliably differentiate between other SGS subtypes. Further, while there is emerging evidence supporting distinct pathophysiology of ANCA‐negative and idiopathic SGS, our cohort's clinical diagnoses likely introduced error. Another limitation is our cross‐sectional design; almost all iSGS patients in our study had a single CRP value at presentation. While a single CRP value does not predict overall aggressiveness of SGS, we were unable to assess if trending CRP in individual iSGS patients could indicate inflammatory phases of SGS with rapid progression or response to steroid injection [25]. This could potentially identify critical times for intervention via pathological activity, instead of overall symptom progression and peak expiratory flow. A prospective study with clearly defined timepoints for trending CRP could more completely assess its potential value in SGS.

## 5 Conclusion

This study characterized the value of serum CRP at presentation in patients with idiopathic and GPA‐related subglottic stenosis. CRP may differentiate iSGS and GPA‐SGS, as significant elevation is associated with GPA‐SGS. A single CRP value does not predict disease aggressiveness when quantified by mean surgery‐free interval. Future work could include trending serum CRP in iSGS patients and comparing it to the baseline established in this study.

## Conflicts of Interest

The authors declare no conflicts of interest.

## Data Availability

The data that support the findings of this study are available on request from the corresponding author. The data are not publicly available due to privacy or ethical restrictions.
